# Transglutaminase binding fusion protein linked to SLPI reduced corneal inflammation and neovascularization

**DOI:** 10.1186/1471-2415-15-12

**Published:** 2015-02-04

**Authors:** Juan P Salica, Diego Guerrieri, Paulo Maffia, Juan O Croxatto, H Eduardo Chuluyan, Juan E Gallo

**Affiliations:** Nanomedicine & Vision Group, Faculty of Biomedical Sciences, Austral University, Juan Domingo Perón, 1500 1629 Pilar, Buenos Aires, Argentina; Department of Ophthalmology, Austral University Hospital, Pilar, Buenos Aires, Argentina; Laboratory of Immunomodulators, School of Medicine, Cefybo (Conicet-University of Buenos Aires), Buenos Aires, Argentina; Department of Ocular Pathology, Fundación Oftalmológica Argentina “Jorge Malbrán”, Buenos Aires, Argentina

**Keywords:** SLPI, Cementoin, Transglutaminase, Corneal neovascularization, Angiogenesis, NFkB, Corneal inflammation, Alkali injury

## Abstract

**Background:**

To study the effect of topical administration of a fusion protein (PF-MC) made up of N-terminal portion of the protease inhibitor Trappin-2 (which is a substrate of transglutaminasa-2) and SLPI (protein with anti-inflammatory, anti-bacterial and anti-viral ability), in an animal model of corneal inflammation and angiogenesis.

**Methods:**

An alkali injury was produced with a filter paper of 3 mm with 1 N NaOH during 40 seconds on the right cornea of 36 male Sprague Dawley rats, under general anesthesia. Animals were divided into three groups according to treatment. Group 1 was treated with 10 ul of PF-MC (200 ug/ml; n = 12), Group 2, with 10 ul of SLPI (200 ug/ml; n = 12) and Group 3 was treated with buffer (10 ul; n = 12) topically administered four times a day for up to 7 days. Half of the animals were sacrificed at day 3 before making a re-epithelialization time analysis with fluorescein staining at 18 and 24 hours. In the remaining animals corneal opacity was studied and digital photographs were taken at day 7 before doing euthanasia. Eyes were processed for histology and immunofluorescence.

**Results:**

Corneal ulcerated area was significantly lower in PF-MC treated animals compared to SLPI and buffer-treated animals at 18 hours and 24 hours postinjury. A clear cornea and fundus red reflex was only found among PF-MC treated animals. Histological analysis revealed a stratified corneal epithelium with at least three layers in all PF-MC animals at day 7. In this group there was a reduced number of PMNs in the corneal stroma at 3 and 7 days of follow-up. Besides, corneal neovascularization was much more extended in SLPI and Buffer animals than in animals treated with PF-MC.

**Conclusions:**

The binding of SLPI with Cementoin to transglutaminase seems to be an effective strategy to treat corneal inflammation and angiogenesis.

**Electronic supplementary material:**

The online version of this article (doi:10.1186/1471-2415-15-12) contains supplementary material, which is available to authorized users.

## Background

The use of steroids in corneal inflammatory diseases is quite common [1–3]. Corticoids chronic use can cause ocular side effects as glaucoma and cataracts and affect corneal epithelialization [4–6]. Because of these problems other anti-inflammatory drugs are being tested, such as the physiological serine protease inhibitors SLPI (secretory leucocyte protease inhibitor) and Elafin. These drugs neutralize the activity of polymorphonuclears (PMNs) elastase but also inhibit the translocation of the transcription factor NFkB (nuclear factor kappa-light-chain-enhancer of activated B cells) to the core hindering the activation of pro-inflammatory genes [7].

The proteolysis inhibitory action plays an important role in the control of tissue damage produced by proteases in the inflammatory sites. A strict balance between proteases and the inhibitory effect is indispensable in wound healing and in inflammatory disorders [8]. Unbalance occurs in various inflammatory diseases as fibrocystic disease, chronic bronchitis, emphysema associated to smoking and ulcers in inflammatory bowel disease [9–12].

SLPI is a low molecular weight protein that was identified as protease inhibitors in mucosal secretions. In recent years, our laboratory investigated the role of SLPI in several inflammatory processes, such as orchitis autoinmmune and tuberculosi [13]. Therapies with SLPI are not easy to maintain because of SLPI short half-life in serum [7]. In order to improve the bioactive capabilities of SLPI some modifications at the molecular level were performed recently in our laboratory (unpublished results).

Transglutaminase (TG) is a group of 9 enzymes that catalyze the formation of an isopeptide bond between a free amine group and the acyl group at the end of the side chain of protein- or peptide-bound glutamine. These enzymes are involved in processes such as inflammation, re-epithelialization, neovascularization and synthesis of fibrous extracellular matrix. TG2 is located in the cell nucleus, the cytoplasmic matrix and membrane compartments [14]. TG2 overexpresses at the inflammatory site and activates PLA2, NFkB and MAPK stimulating the inflammatory response. TG2 has been studied on the eye and its relations with different pathologies. It is involved in the pathophysiology of wound healing, pterygium, dry eye and allergic conjunctivitis, among others [15, 16]. Therefore, there is a strong hypothesis that inhibiting TG2 could be beneficial for the cornea at least at some point of the inflammatory process.

Combining the concepts previously exposed, the SLPI gene was fused to the N-terminal portion of the protease inhibitor Trappin-2 (cementoin), and expressed in a recombinant form as a fusion protein, designated PF-MC. This fusion protein was able to anchor at the different sites where the TG2 is expressed. Immobilized in this way, it allows the SLPI molecule to act in a localized fashion, and probably to increase the half-life of the protein. Therefore, the molecule is retained in the site where the transglutaminase enzyme is present, particularly through TG2 [17]. In this way, the biological active agent has a longer half-life in the specific site of therapy and a lower plasmatic degradation and would silence the TG2 pro-inflammatory activity [18]. This mechanism is especially useful for topical eye treatments because blinking accelerates drugs washed-up.

Corneal alkali injury cause very important damage to the eye. There is a great inflammation and release of collagenases as well as proteases that severely damage the corneal tissue [19]. The inhibition of the NFkB pathway, which is activated by most inflammatory cytokines, will probably reduce the magnitude of the inflammatory process, improving wound healing. For this purpose, we aimed at evaluating the topical effect of the above mentioned fusion protein in a rat model of corneal alkali injury. Results shown herein are promising.

## Methods

### Experimental design

Thirty-six male Sprague–Dawley rats (12 weeks old) were provided by CNEA (Comisión Nacional de Energía Atómica, Buenos Aires, Argentina). The animals were initially examined and screened for any preexisting ophthalmic lesion. General anesthesia was induced using isoflurane and then an alkali injury was produced with a filter paper of 3 mm with 1 N NaOH during 40 seconds on the central part of the right cornea [20]. Then, the ocular surface was gently washed with 5 ml of saline solution with 35G cannula. Animals were divided into three groups according to the treatment: 1) PF-MC (200 ug/ml); 2) SLPI (200 ug/ml), 3) Elution Buffer. Therapy was applied topically 10 ul four times daily. The first dose was administered 30 minutes after the lesion was caused. Six animals of each group were euthanized at 3 and 7 days post lesion, respectively. All experimental procedures were done under the ARVO Statement for the Use of Animals in Ophthalmic and Vision Research and approved by the Austral University’s Animal Care and Use Committee.

### Preparation of PF-MC

The expression and purification of recombinant protein PF-MC protein was performed from E. coli BL21-Codon Plus-RIL (Stratagene, Darmstadt, Hessen, Germany). The bacterial pellets were lysed by ultrasound and centrifuged. The soluble fraction was recovered and added to a column of Nickel-nitrilotriacetic acid (Ni-NTA) (Qiagen GmbH, Hilden, Westphalia, Germany), allowing the binding of the tail of histidines present in the protein. The PF-MC protein retained in the column was eluted by adding 2 ml of 50 mM NaH2PO4, 300 mM NaCl, 250 mM imidazole; pH = 8 (elution buffer 250). To reduce the concentration of imidazole in the samples of proteins, the eluates were subjected to a dialysis in phosphate buffer having a final concentration of 2.5 mM. The dialysis was performed overnight at 4°C, and subsequently the protein was recovered and aliquoted as well as the dialysis buffer (to be used as controls in the assays). After the purification and in order to remove the residual bacterial LPS polymyxin B-agarose agar was washed with dialysis buffer. The protein PF-MC was recovered by centrifugation of the agar. The quantification of protein concentration was performed with a MicroBCA (Pierce, USA) kit according to the manufacturer’s instructions.

### Evaluation of corneal re-epithelialization time

Corneal re-epithelialization following alkali burn was studied by fluorescent dye staining of the cornea. Areas with epithelial defect retain staining and absence of stained area indicates complete re-epithelialization. Six eyes of each group were stained with fluorescein dye and examined every 6 hours under an operating microscope (Zeiss S4, Germany) adding cobalt blue light. Digital pictures were taken at 18 and 24 hours of the injury using a Digital Camera (20.1 megapixel; Nikon, Coolpix S3500, Japan) with a Microscope Ocular Adaptor. Pixels of the stained area were measured with Adobe Photoshop after standardizing the size of the corneas. Percentage of ulcerated cornea was calculated. The results were presented as mean ± standard error of the mean. To study the statistical difference between groups, we applied Kruskal-Wallis test.

### Evaluation of corneal opacity

The corneal opacity was evaluated using a slit-lamp biomicroscope (Haag-Streit, Germany) at day 7. Findings were classified according to Fantes *et al*. [21]. Briefly, Grade 0: complete clear cornea without any trace of haze. Grade 0.5: a faint haze detectable only by oblique illumination. Grade1: mild haze but not interfering with visibility of iris details. Grade 2: more prominent haze with mild obscuration of iris details. Grade 3: opacity of moderate density easily detectable under direct illumination with partial obscuration of iris details. Grade 4: complete opacity with no visibility of structures in the anterior chamber. The results are presented as mean ± standard error of the mean. To study the statistical difference between groups, we applied Kruskal-Wallis test. We studied and analyzed corneas with the presence of abscess.

### Histological examination

Animals were euthanized at day 3 and 7. The eyeballs were extracted and the cornea dissected. Then, the cornea was processed for cryosections of 15 microns thickness and stained with hematoxyline and eosine. Four sections of each cornea were examined under an Eclipse Microscope (Nikon, Tokyo, Japan). The evaluation of the cornea included the morphology of cells and number of layers of the corneal epithelium; the count of cells in the stroma; the identification and topographical localization of neovessels. The analysis was carried out masked by two examiners. The difference between the two examiners was tested.

**Figure 1:**
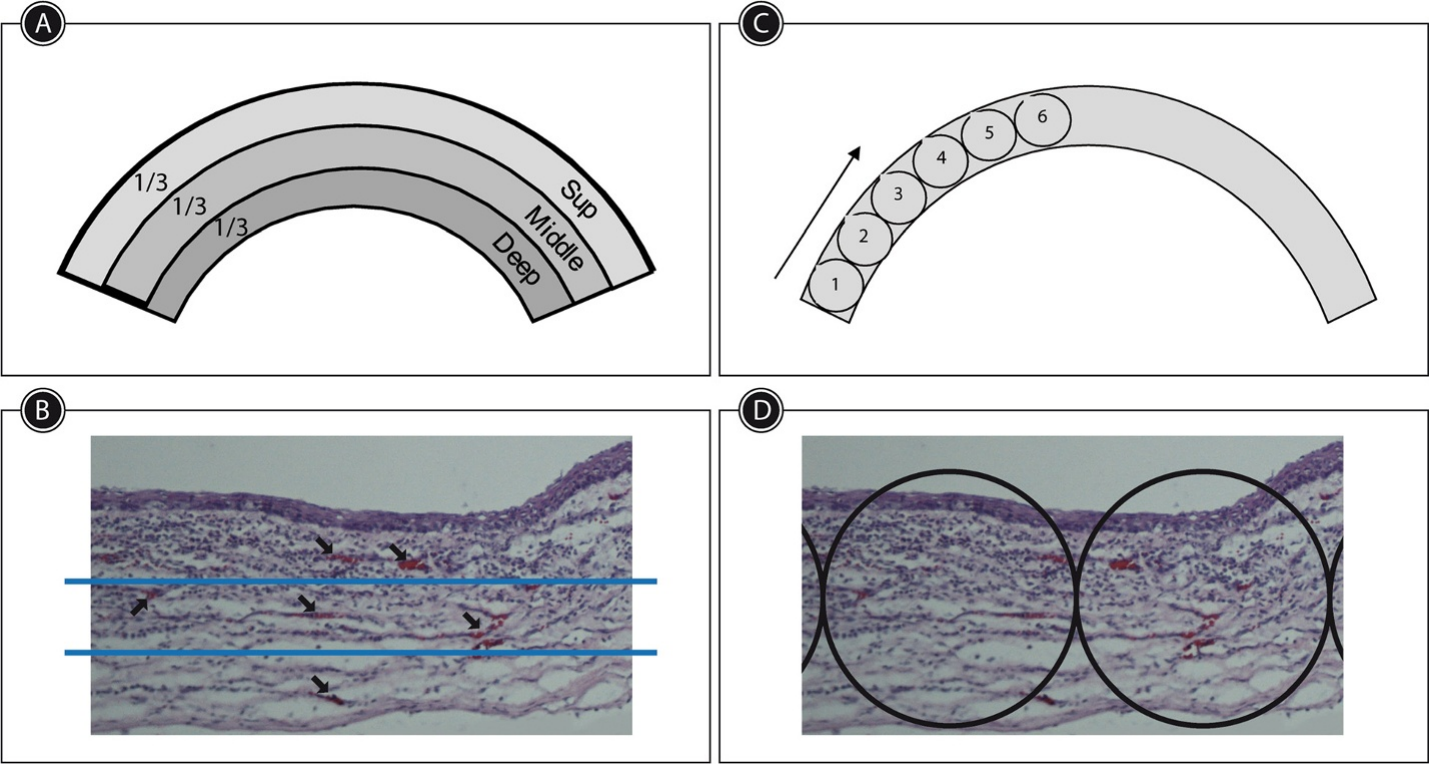
**Method used to evaluate the corneal neovascularization (CNV). A**: The Corneas were divided into thirds from epithelial surface to endothelium. **B**: Sample of a cornea coloured with hematoxylin and eosin (h&e). Blue lines show the virtual division in thirds of the corneas. The vessels identified in each field (black arrows) counted as positive. **C**: Schematic cornea showing 40X fields from the periphery to the center. **D**: Sample of a cornea stained with h&e, black circles marked the 40X fields used to measure CNV extension.

Neovascularization analysis. The cornea was schematically divided into thirds from epithelial surface to endothelium in order to classify neovessels as superficial, medial and deep (Figure 1A-B). The extension of neovascularization was recorded according to the number of microscopic fields of X40 magnification containing vessels from the periphery to the center of the cornea (Figure 1C-D).

### Immunofluorescense analysis

Rats were euthanized 7 days postinjury and the corneas were excised, fixed in 4% paraformaldehyde for 6 hours (Sigma-Aldrich, St Louis, MO). They were then immersed for cryoprotection in 4 concentrations of glucose (5%, 7.5%, 10% and 20% overnight). The eyes were embedded in Cryoplast (Biopack, Argentina) and frozen with liquid nitrogen. The 15-micron thick cryosections were first incubated overnight at 4°C with anti-mouse polyclonal antibody against vascular endothelial growth factor (VEGF) (1:500) (Santa Cruz Biotechnology Inc., 2145 Delaware Avenue Santa Cruz, CA, USA). Sample preparations were incubated for 30 minutes with donkey anti-mouse IgG-R (SC-2300 Santa Cruz Biotechnology Inc., 2145 Delaware Avenue Santa Cruz, CA, USA) and mounted with glycerin. The evaluation was performed using immunofluorescence microscope (Nikon Eclipse, Tokyo, Japan).

### Statistical analysis

The statistical analysis was performed with Prism 6.0 software. To study the statistical difference between groups, we applied Kruskal-Wallis test. The significance level was set at p < 0.05.

## Results

### Evaluation of corneal re-epithelialization time

**Figure 2:**
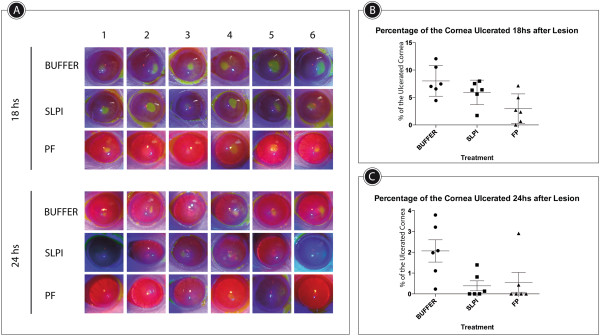
**Corneal re-epithelialization time. A**: Digital pictures of alkali-injured rat corneas treated with PF-MC, SLPI and Buffer, stained with fluorescein under cobalt blue light at 18 and 24 hs. **B**-**C**: Percentage of cornea with epithelial defect (including ulceration) at each healing interval. The results are expressed with M ± SEM.

At 18 hours after lesion, in the group of PF-MC treated animals there was the first cornea with complete re-epithelialization following alkali burn. At 24 hours postinjury this group and the SLPI group had 4 and 3 complete re-epithelialized corneas respectively. At this time re-epithelialization was still incomplete in all corneas of buffer treated animals (Figure 2). The rate of corneal ulcerated area was significantly lower in PF-MCP treated animals compared to that seen in SLPI and buffer treated animals 18 hours postinjury (Figure 2B). The difference between PF-MC and Buffer groups remained similar 24 hours after injury. At this time interval the comparison between SLPI and PF-MC treated animals did not show a statistically significant difference (Figure 2C).

### Macroscopic analysis

**Figure 3:**
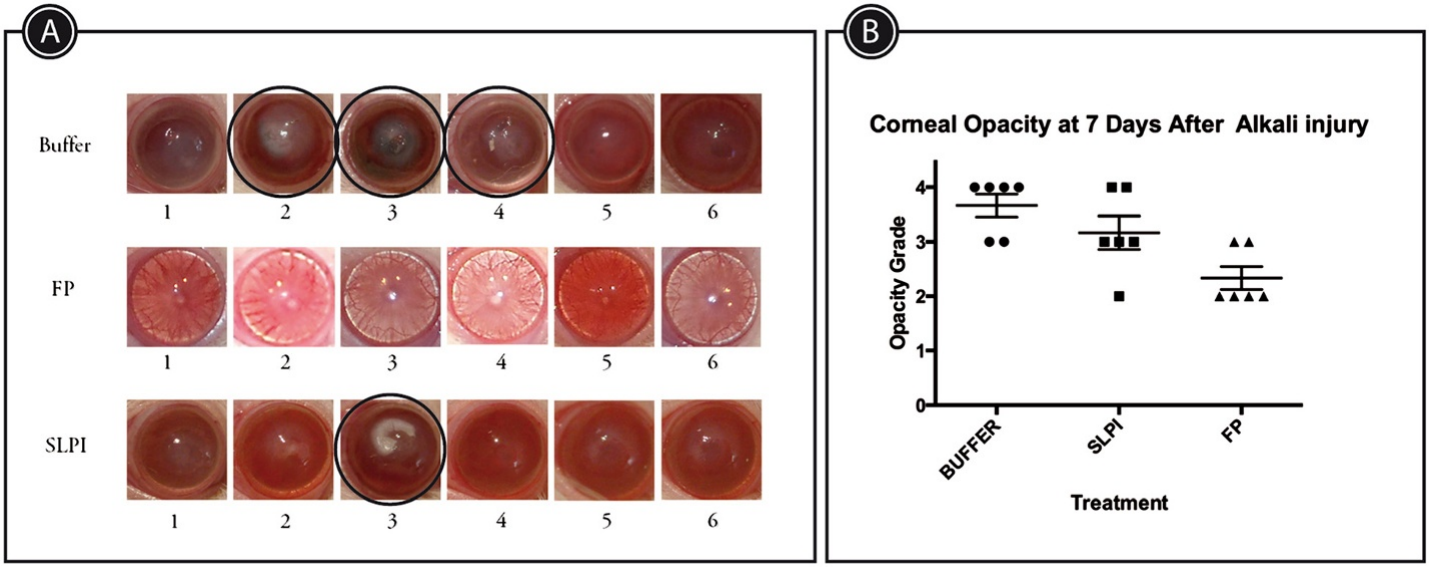
**Clinical digital photographs of rat corneas at day 7 after alkali-injury treated with PF-MC, SLPI or Buffer. A**: Macroscopic observation shows presence of corneal stromal abscesses marked with black circle in SLPI and Buffer groups. **B**: Scatter dot plot showing corneal opacity grade of each treated group, classified according to Fantes *et al*.

Corneal clarity was only seen in animals treated with fusion protein at 7 days of follow-up. We observed a fundus red reflex and lack of corneal abscess in all PF animals (Figure 3A). SLPI and Buffer groups showed opaque light reflex at the fundus red reflex test. In addition, corneal abscess was found in one SLPI animal and in 3 Buffer animals. In these groups there were different degrees of corneal opacity.

### Corneal opacity

Fluorescent dye staining of the cornea six hours after alkali injury showed severe destruction of the 3 mm central epithelium in the three groups. At day 7, there was a significant difference in the corneal haze score between the three groups (P = 0.0137). PF-MC treated eyes (2.33 ± 0.210) regained the corneal transparency and allowed clear visualization of the anterior chamber structures through cornea. Not all the corneas treated with Buffer (3.667 ± 0.210) or SLPI (3.167 ± 0.307) regained transparency (Figure 3B).

### Corneal epithelium

Three days postinjury. PF-MC animals had a stratified epithelium with three layers except in one animal. The degree of stratification was greater than that seen in the other animal groups (Additional file 1: Table S1A).

**Figure 4:**
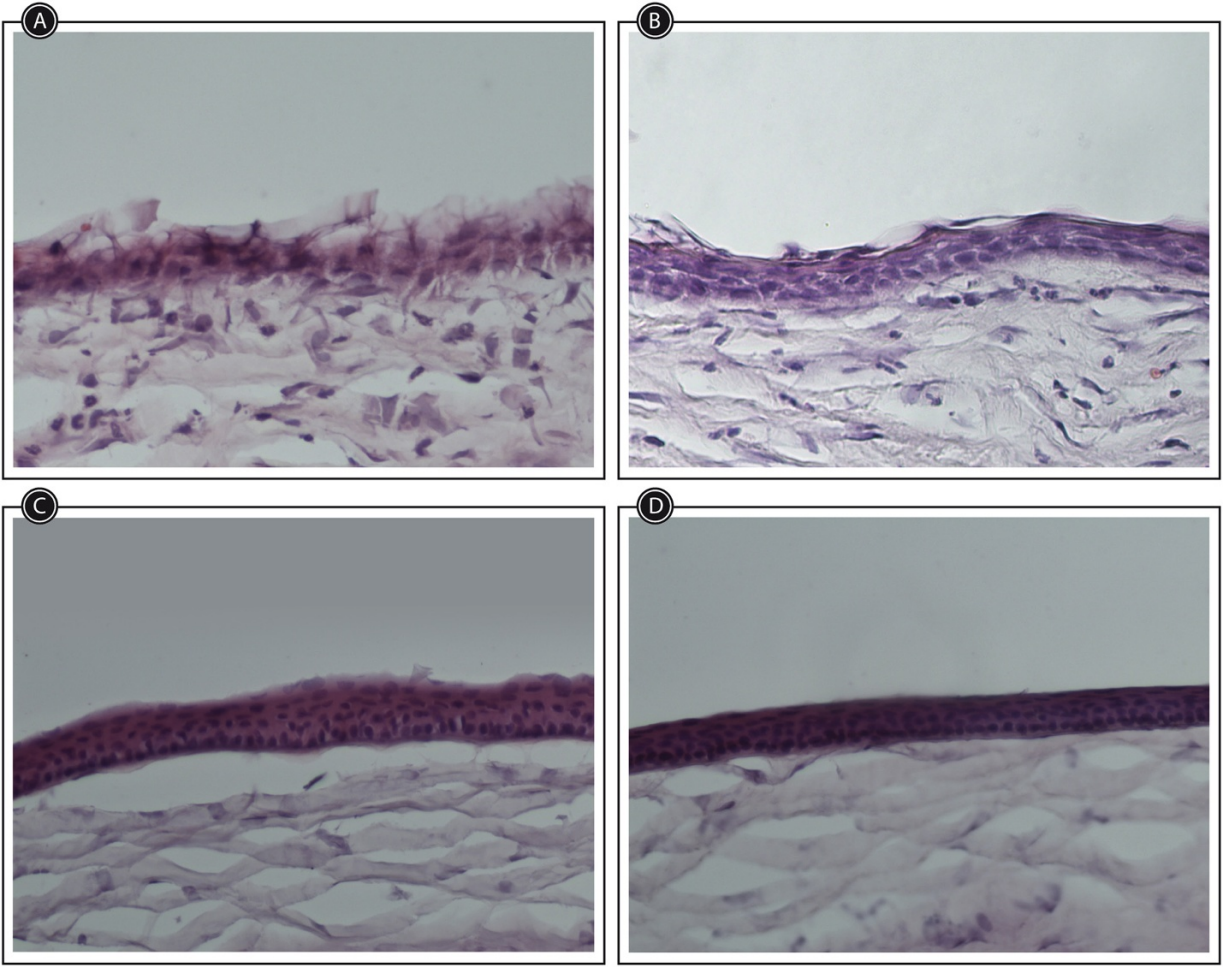
**Corneal epithelialization at day 7 after alkali-injury treated with PF-MC, SLPI and Buffer.** Histology of corneas stained with h&e. **A**: Buffer. **B**: SLPI. **C**: PF-MC. **D**: Healthy control.

Seven days postinjury. Histological analysis revealed a stratified corneal epithelium with at least three layers in all PF-MC (Additional file 1: Table S1C). The morphology of cells looked rather normal in this group of animals. However, the other groups of animals disclosed a thin corneal epithelium with one or two layers. In some cases there was an erratic corneal healing (Figure 4).

### Cell count of the corneal stroma

Three days post injury. PF-MC animals had a reduced number of PMNs and total cells than that observed in SLPI and Buffer animals (Additional file 2: Table S2A).

**Figure 5:**
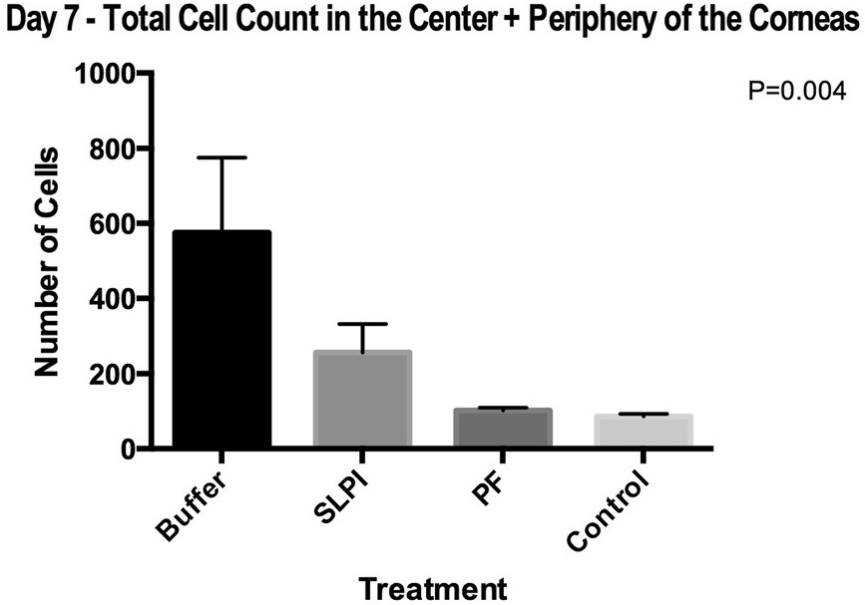
**Total cells count of corneas at day 7 post-injury, treated with PF-MC, SLPI or Buffer.** Count of PMN in one 40X field at the center of the cornea plus the PMN in one 40X field al the periphery of the cornea.

Seven days post injury. The number of cells counted in the corneal stroma was much lower in animals treated with PF-MC than in the other treated groups (Figure 5). Similar differences were found whether the center or periphery of the cornea was considered (Additional file 3).

**Figure 6:**
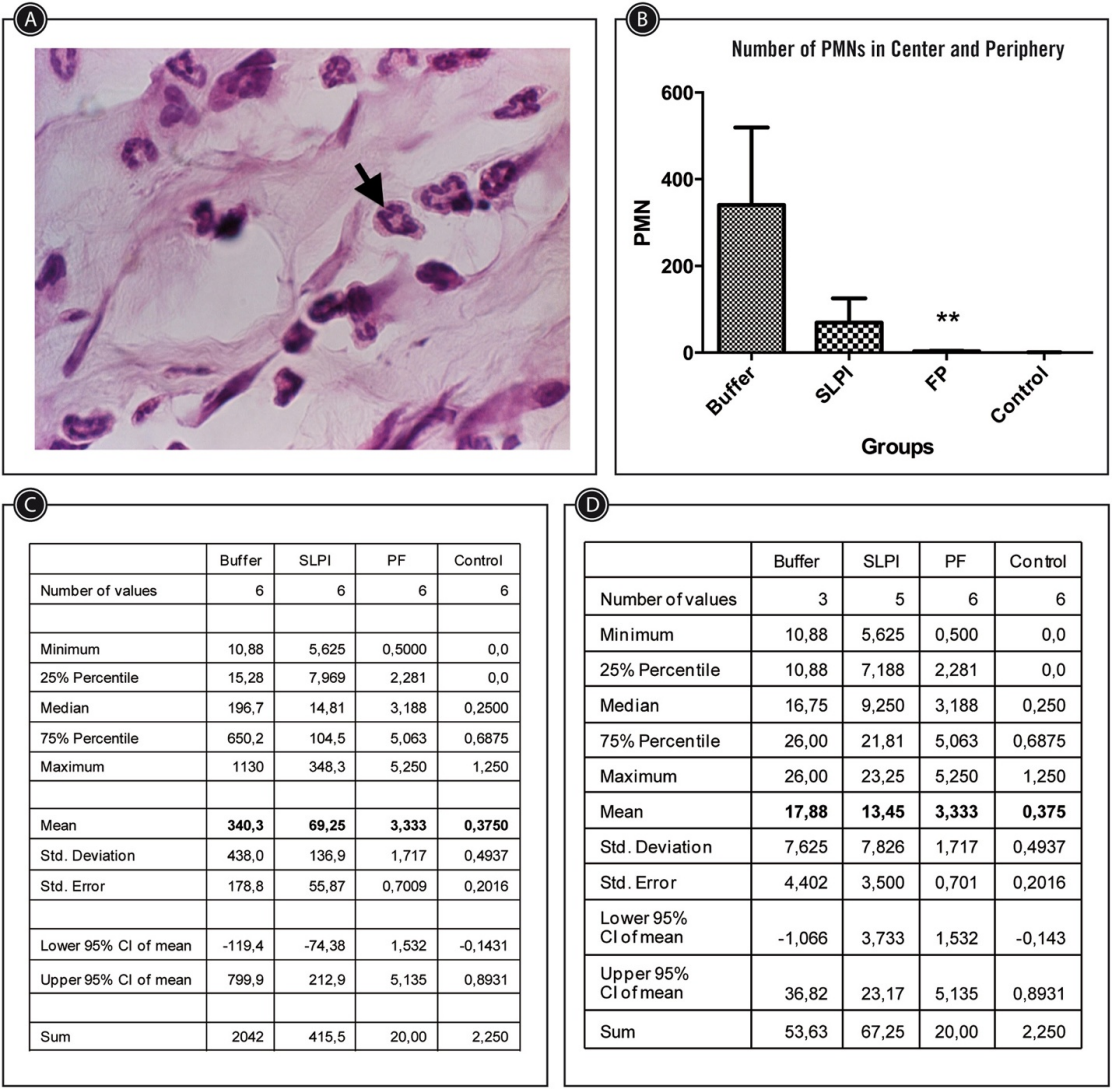
**Polimorphonuclear count at the center and periphery of corneas at day 7 after alkali-injury treated with PF-MC, SLPI or Buffer. A**: Microscopic picture (40X). Black arrow indicates a PMN cell. **B**: Column chart of number of PMN in a 40X visual field at the center plus a field at the periphery. **C**: Statistic table of the PMN count at the center and periphery. **D**: Statistic table of PMN count at the center and periphery excluding the cases with stromal abscess.

The quantity of PMN cells was significantly reduced in animals under PF-MC treatment compared to other animals (Figure 6). The exclusion of cases that had corneal abscess (severe corneal infection with opacity) did not modify the degree of differences within groups.

### Corneal neovascularization

**Figure 7:**
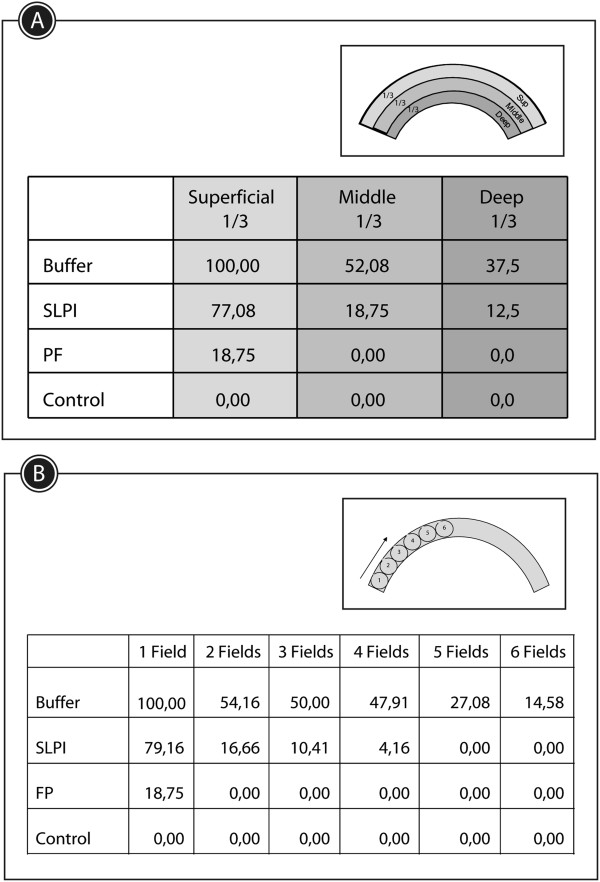
**Results of corneal neovascularization (CNV) analysis. A**: Table showing percentage of rats of each treated group with CNV at different depth of the cornea. **B**: CNV extension at day 7. Percentage of animals of the studied groups that showed neovessels at each field from the periphery to the center.

At 7 days postinjury PF-MC animals had only superficial neovessels while the other animals also had vessels in the middle third and deep third of the cornea (Figure 7A). Only animals that received buffer showed neovessels growth into the six fields of recording (Figure 7B). Interestingly, blood vessels among PF-MC animals were found in only one field.

At 3 days postinjury there were some neovessels in the cornea of SLPI and buffer groups. PF-MC animals showed very few corneal neovessels.

### Immunofluorescence

**Figure 8:**
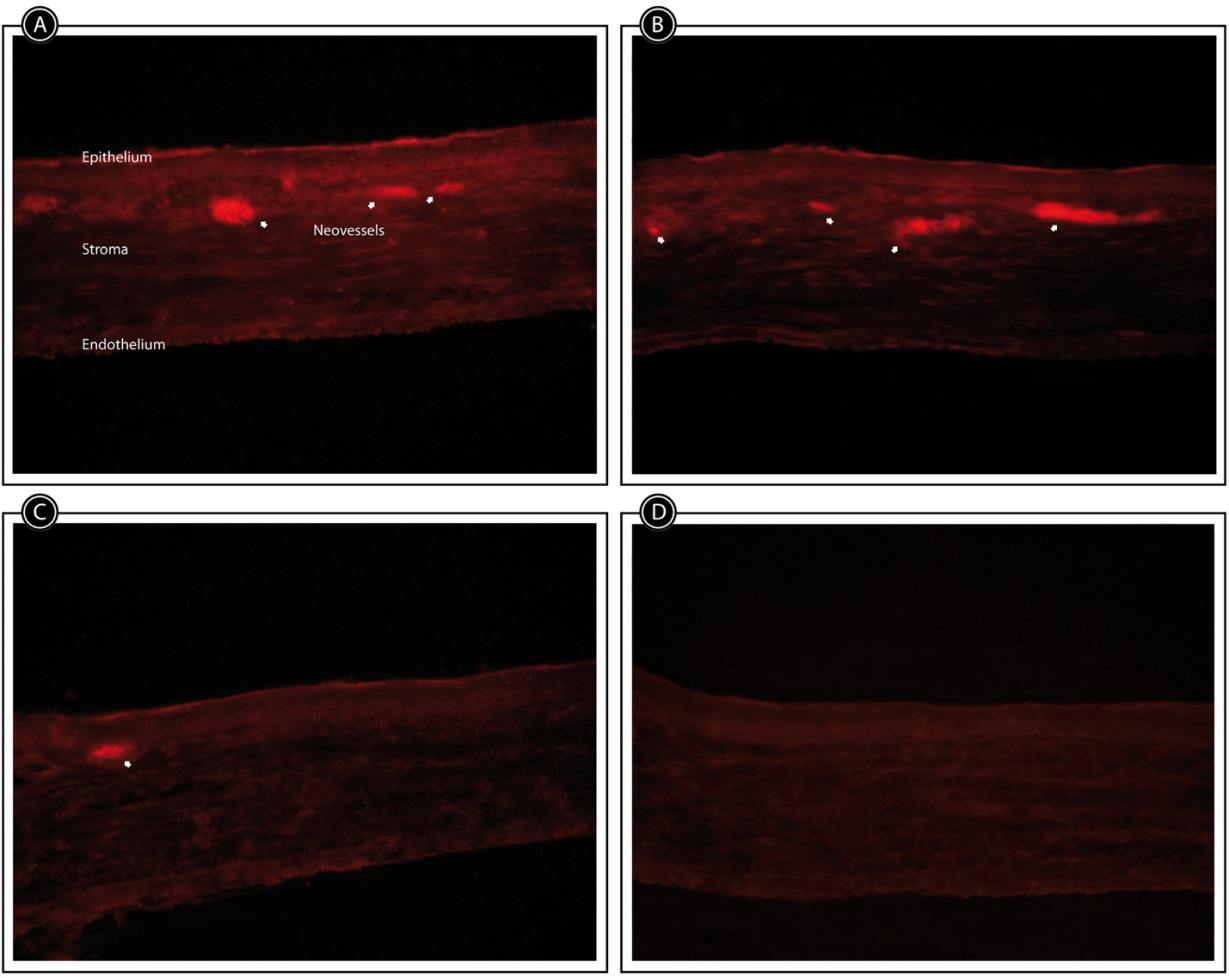
**Representative VEGF immunofluorescence staining of corneal sections at day 7 after alkali-injury treated with PF-MC, SLPI or Buffer.** VEGF revels in red the presence of neovessels in the cornea of the treated rats, indicated by white arrows. **A**: Buffer. **B**: SLPI. **C**: PF-MC. **D**: Healthy control.

Staining of VEGF was much more extended in the cornea treated with buffer than in the other groups of animals at day 7 (Figure 8).

## Discussion

We have analyzed the topical effect of a new fusion protein made up of SLPI and N-terminal portion of the protease inhibitor Trappin-2 in a rat model of corneal alkali injury. Animals treated with the PF-MC had less corneal inflammation and neovascularization than that found in rats treated with SLPI or buffer.

The characteristics and potential effects of the protein fusion have been previously reported. Cementoin (a terminal of Elafin) brings SLPI the capacity to unite to other molecules present in the intercellular interstitium by transglutamination. The SLPI is retained in the site where the transglutaminase enzyme is present [18].

All the animals treated with the PF-MC showed corneal clarity and retinal red reflex at seven days of follow-up. No corneal abscess was observed. It is known that SLPI has antimicrobial effect [7, 13, 22]. The relative higher effect of SLPI in the PF-MC might have contributed to prevent the development of a serious corneal abscess as we observed in the other groups of animals.

Corneal neovascularization was found within the three groups of animals at 7 days postinjury. Nevertheless, the extension of angiogenesis was much lower in the PF-MC group and vessels were only seen in the superficial part of the cornea. It is known that neovessels develop in the middle to the anterior stroma of the cornea [23]. We observed the presence of vessels in the deeper third of the cornea in the cases of severe inflammation. The findings of this study suggest the possibility of a direct relationship between inflammation and depth of hemangiogenesis in the corneal stroma.

The histological examination was carried out by two investigators, double blinded, to avoid bias. The difference between them was tested but it was not significant. The mean of both examiners was included in our research study. The number of total cells and PMNs in the corneal stroma was much more reduced in PF-MC animals compared to animal groups treated with SLPI or Buffer. The greater extent of VEGF immunoreactivity in corneas of buffer treated animals compared to the other groups correlated with the histological findings.

## Conclusion

Topical treatment of the fusion protein made up of SLPI + Cementoin showed good anti-inflammatory and antiangiogenic properties in a rat model of corneal alkali injury. New applications of this fusion protein are being tested in other ocular diseases.

## Electronic supplementary material

Additional file 1: **Statistical Analysis of Epithelium Layers Count at one 40X field in sections of healthy and alkali injured corneas treated with PF-MC, SLPI or Buffer.** A: Count in the Center of the Corneas at day 3. B: Count in the Periphery of the Corneas at day 3. C: Count in the Center of the Corneas at day 7. D: Count in the Periphery of the Corneas at day 7. (DOC 328 KB)

Additional file 2: **Statistical Analysis of Polymorphonuclear Neutrophils Count at one 40X field in sections of healthy and alkali injured corneas treated with PF-MC, SLPI or Buffer.** A: Count in the Center of the Corneas at day 3. B: Count in the Periphery of the Corneas at day 3. C: Count in the Center of the Corneas at day 7. D: Count in the Periphery of the Corneas at day 7. (DOC 333 KB)

Additional file 3: **Statistical Analysis of Total Cell Count at one 40X field in sections of healthy and alkali injured corneas treated with PF-MC, SLPI or Buffer.** A: Count in the Center of the Corneas at day 3. B: Count in the Periphery of the Corneas at day 3. C: Count in the Center of the Corneas at day 7. D: Count in the Periphery of the Corneas at day 7. (DOC 338 KB)

## Abbreviations

CNEA: Comisión Nacional de Energía Atómica
LPS: Lipopolysaccharide
MAPK: Mitogen-activated protein kinases
NFkB: Nuclear factor kappa-light-chain-enhancer of activated B cells
PLA2: Phospholipases A2
PMN: Polymorphonuclears cells
SLPI: Secretory leucocyte protease inhibitor
TG: Transglutaminase
TG2: Transglutaminase-2
VEGF: Vascular endothelial growth factor.
