# Supplementary figures and images for: Transglutaminase binding fusion protein linked to SLPI reduced corneal inflammation and neovascularization
Source: BMC Ophthalmol. 2015 Feb 4;15:12. doi: 10.1186/1471-2415-15-12 (PMC4603969; doi:10.1186/1471-2415-15-12)

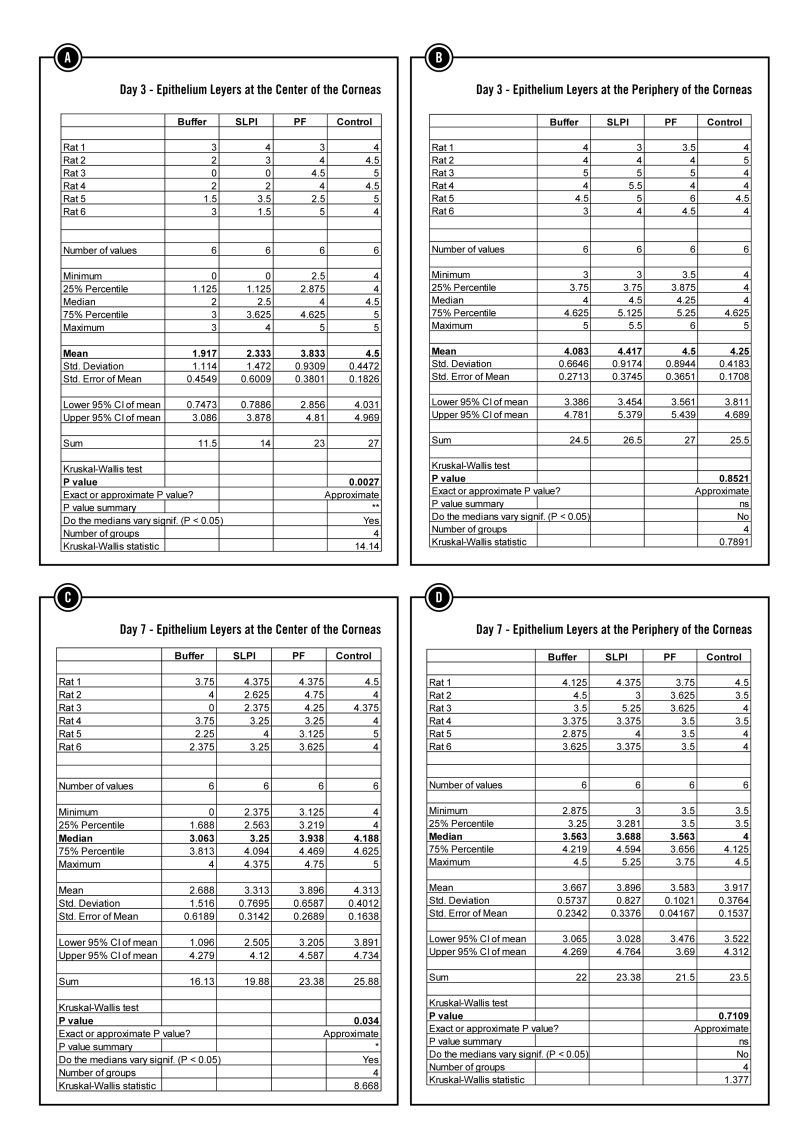

Supplement: Supplementary file 1 — Additional file 1: Statistical Analysis of Epithelium Layers Count at one 40X field in sections of healthy and alkali injured corneas treated with PF-MC, SLPI or Buffer. A: Count in the Center of the Corneas at day 3. B: Count in the Periphery of the Corneas at day 3. C: Count in the Center of the Corneas at day 7. D: Count in the Periphery of the Corneas at day 7. (DOC 328 KB) [file 12886_2014_538_MOESM1_ESM.doc]

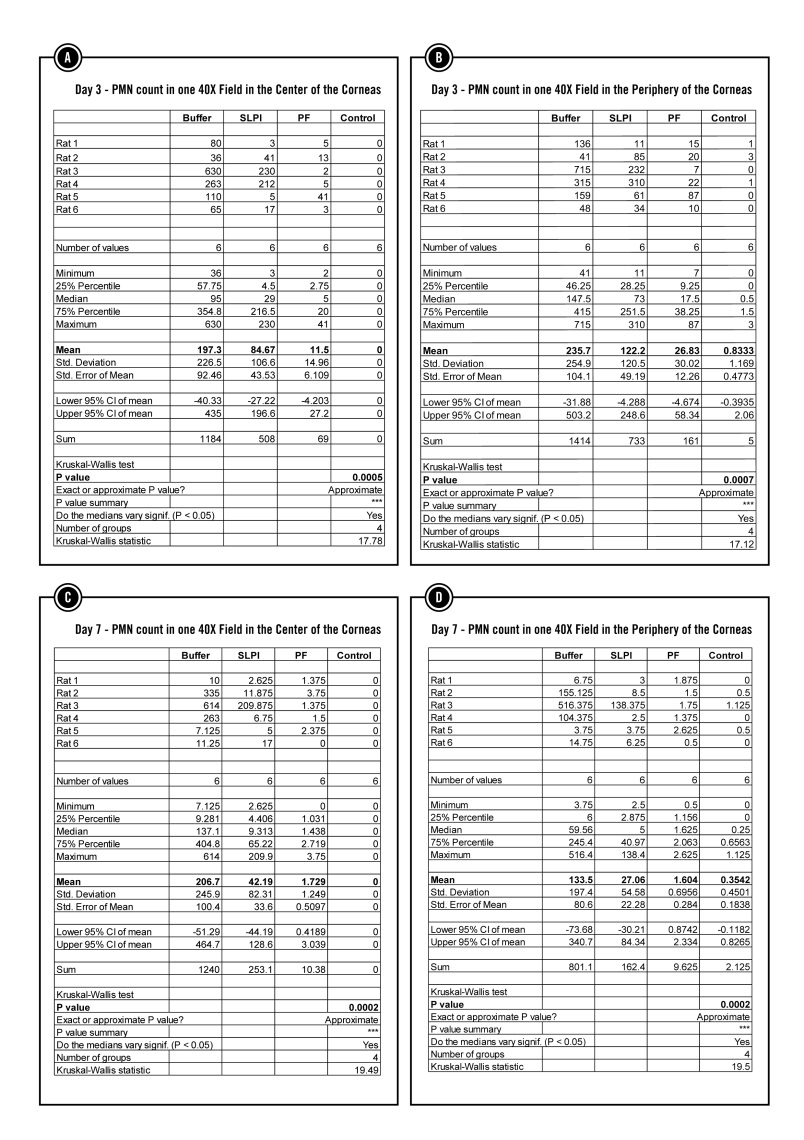

Supplement: Supplementary file 2 — Additional file 2: Statistical Analysis of Polymorphonuclear Neutrophils Count at one 40X field in sections of healthy and alkali injured corneas treated with PF-MC, SLPI or Buffer. A: Count in the Center of the Corneas at day 3. B: Count in the Periphery of the Corneas at day 3. C: Count in the Center of the Corneas at day 7. D: Count in the Periphery of the Corneas at day 7. (DOC 333 KB) [file 12886_2014_538_MOESM2_ESM.doc]

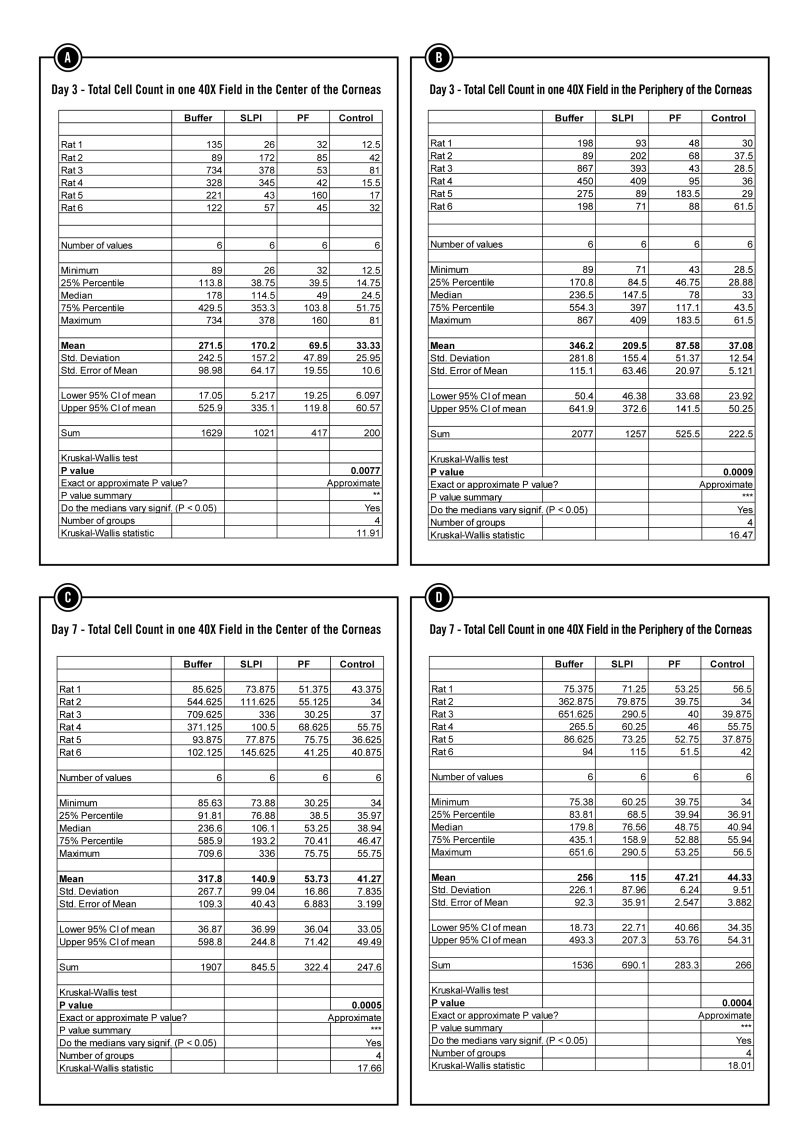

Supplement: Supplementary file 3 — Additional file 3: Statistical Analysis of Total Cell Count at one 40X field in sections of healthy and alkali injured corneas treated with PF-MC, SLPI or Buffer. A: Count in the Center of the Corneas at day 3. B: Count in the Periphery of the Corneas at day 3. C: Count in the Center of the Corneas at day 7. D: Count in the Periphery of the Corneas at day 7. (DOC 338 KB) [file 12886_2014_538_MOESM3_ESM.doc]
